# An Introduction to Social Media for Scientists

**DOI:** 10.1371/journal.pbio.1001535

**Published:** 2013-04-23

**Authors:** Holly M. Bik, Miriam C. Goldstein

**Affiliations:** 1 UC Davis Genome Center, University of California Davis, Davis, California, United States of America; 2 Scripps Institution of Oceanography, University of California San Diego, San Diego, California, United States of America; 3 California Sea Grant, La Jolla, California, United States of America

## Abstract

Online social media tools can be some of the most rewarding and informative resources for scientists—IF you know how to use them.

*Online social media tools can be some of the most rewarding and informative resources for scientists—IF you know how to use them.*

In many ways, the fast-paced evolution of the internet parallels the move toward “big data” in science. In less than a decade, online tools have exploded in popularity and witnessed rapid expansion (Figure 1), with an increasing number of scientists now looking to take advantage of these web-based resources (see Box 1 and Table 1 for an overview and comparison of existing tools). Social media portals in particular undergo regular reinvention and transformation, with different tools becoming popular for different populations [1]. Although a number of guides exist online, many researchers still feel overwhelmed and hesitant toward the virtual world, lacking sufficient information and guidance through formal scientific channels such as peer-reviewed journals. To better familiarize researchers with existing internet resources, here we discuss prospective benefits that can stem from online science conversations, explain how scientists can efficiently and effectively harness online resources, and provide an overview of popular online tools.

**Figure 1:**
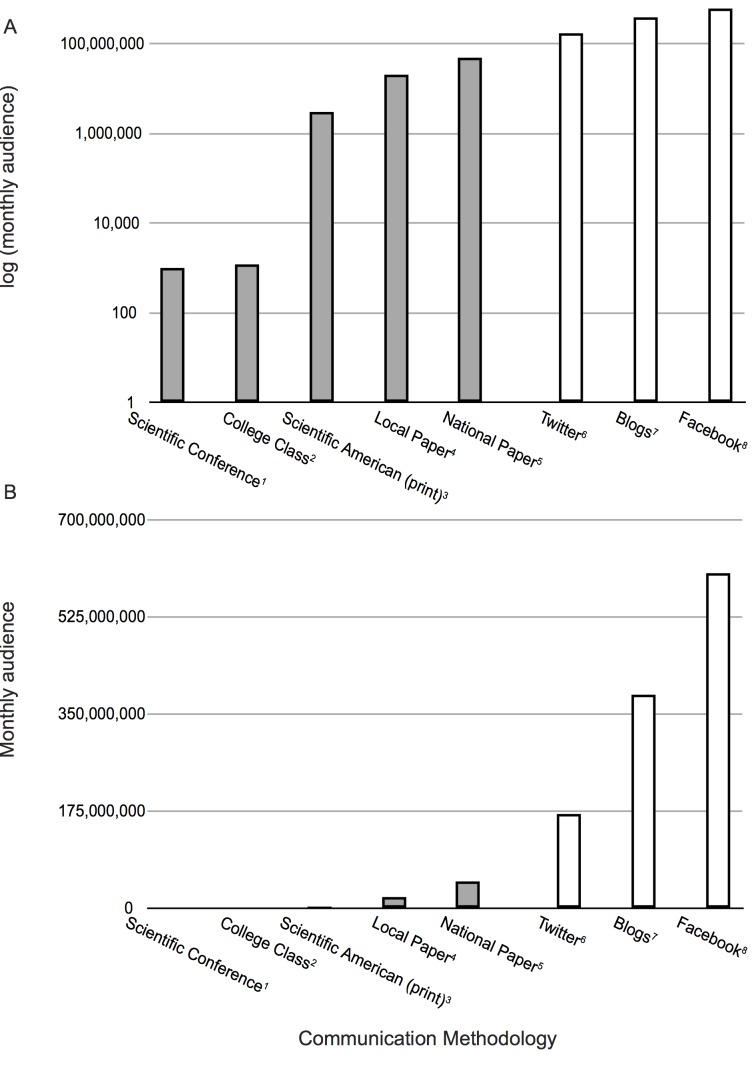
Monthly audience by communication methodology shown on A) log scale and B) linear scale. Filled bars indicate traditional methodologies and unfilled bars indicate online methodologies. Data sources are as follows: 1. estimate; 2. estimate; 3. Scientific American (http://bit.ly/Z0dkaF); 4. San Diego Union-Tribune (http://bit.ly/WusyhV); 5. New York Times (http://bit.ly/14aktDi); 6. Twitter (http://tcrn.ch/146wWsy); 7. Wordpress (http://bit.ly/WVBwDa); 8. Facebook (http://bit.ly/10xUemL). Numbers reflect the potential monthly audience for each medium, and not necessarily the number of users who access a particular content item on that medium. All data accessed on January 22, 2013 and normalized to monthly views.

**Table 1:** Comparison of Online Tools.

Platform	Pros	Cons
Blogs	• Longevity; posts are accessible via search engines• Robust platform for building an online reputation	• Time investment for preparing thoughtful posts• Posts should be disseminated and advertised via other platforms
Twitter	• Low time investment, short posts• Ability to rapidly join in on online conversations• The most current source for breaking news and topical conversation	• Posts are quickly buried under new content• Twitter does not make its archive database accessible to search• Gaining followers can be a slow and difficult process
Facebook	• Established juggernaut in the social media world• Ability to create “groups” and “pages” for a person or cause	• Privacy concerns• Frequent changes to layout, features, and settings
Google+	• Integration with Google tools• Easily manage privacy/visibility by grouping contacts into “circles”	• User base not unique compared to other sites• Users still unsure how to use it

**Box 1. Online Tools & Resources**

**Blogs** - Traditional, long-form online narrative. Wordpress (http://wordpress.com) and Blogger (http://blogger.com) are two of the most popular sites to offer free blog hosting, including easy graphical interfaces for constructing posts and changing blog layouts. If you aren't sure if blogging is for you, or if you only have a few posts in mind, it is reasonable (and common practice) to enquire about a guest post on an established blog with a built-in audience.

**RSS Feeds** - Type of URL that allows users to automatically mine blog/website updates without the need for a web browser. RSS aggregators such as Google Reader are a streamlined and practical way to keep track of new and relevant content. Aggregated RSS feeds can additionally be imported and synced with dedicated apps; for example, MobileRSS is one useful software tool that can be used to access Google Reader feeds on smartphones and tablet devices.

**Apps** - Software used on mobile devices. Apps are especially useful as mobile social networking platforms (e.g., using Twitter, Tumblr, or Facebook apps to post updates while attending scientific conferences), synchronized data repositories (e.g., apps for organizing PDF libraries, address books, or RSS feeds), or as a gateway to connecting people with nature (e.g., popular apps such as Audubon Guides and Starwatch).

**Twitter** (http://twitter.com)- Social networking site that limits posts to 140 characters. Twitter is useful for in-the-moment conversations, customized news streams, and building and maintaining communities. Devices such as hashtags, a phrase beginning with a hash/pound sign (e.g., use #longreads when linking to lengthy online articles), allow users to aggregate tweets according to topic. For example, conference attendees will create a specific hashtag for a particular event, such as #asm2012 for the General Meeting of the American Society for Microbiology that took place in San Francisco (June 16–19, 2012). Tweets incorporating #asm2012 became so popular during the conference that this hashtag was listed as “trending” on the main Twitter homepage—a rare but impressive feat for online scientific discussions.

**Facebook** (http://www.facebook.com) - The most widely used social media site. There are divided opinions about Facebook, and researchers tend to view this site two ways: 1) They create a public profile that may reach a different audience than Twitter or blogs, or 2) They eschew using Facebook for research-related purposes at all, perhaps maintaining private profiles for only their closest friends and family (don't get offended if they don't accept your friend request!).

**Tumblr** (http://www.tumblr.com) - A microblogging site that can publish any type of media very easily and quickly. Users post photos, videos, or short quotes as opposed to long written narratives. Tumblr offers automatic forwarding of new posts to Facebook and Twitter accounts.

**Pinterest** (http://pinterest.com) - A photo-only microblogging site where users define themed “boards” for posting content (e.g., food, art, marine fish). Pinterest is a new and emerging social media site whose user demographics are significantly different from other portals (82% women [15]). “Pins” can also be shared via Facebook and Twitter. Oregon State University's Superfund program maintains a Pinterest board on science communication (http://bit.ly/WbDUHd).

**Storify** (http://storify.com) - A way to aggregate and organize tweets, videos, blog posts, and other media. Storify is especially useful for compiling media on discrete discussions and preserving tweets before they become archived by Twitter. For example, if there is a panel discussion or academic seminar, a Storify can be created that includes live tweets from the audience, videos of the panelists, and links to their publications, websites, and social media profiles.

**Linking communities** - Include Digg (http://digg.com), StumbleUpon (http://www.stumbleupon.com), MetaFilter (http://www.metafilter.com), and more. These are content aggregation sites that recommend new and interesting content to subscribers.

## Research Benefits from an Online Presence

In the age of the internet, social media tools offer a powerful way for scientists to boost their professional profile and act as a public voice for science. Although the type of online conversations and shared content can vary widely, scientists are increasingly using social media as a way to share journal articles, advertise their thoughts and scientific opinions, post updates from conferences and meetings, and circulate information about professional opportunities and upcoming events. Google searches now represent the standard approach for discovering information about a topic or person—whether it be search committees collecting information about faculty candidates, graduate students searching out prospective labs, or journalists on the hunt for an expert source. Consequently, in today's technology-driven world, lack of an online presence can severely limit a researcher's visibility, and runs the risk that undesirable search results appear before desirable ones (however, this scenario is easily rectified; see Box 2). A growing body of evidence suggests that public visibility and constructive conversation on social media networks can be beneficial for scientists, impacting research in a number of key ways.

**Box 2. Advice for New Users**

In academia, there is often a particular stigma attached to online activities. Actively maintaining an online profile and participating in social media discussions can be seen as a waste of time and a distraction from research and teaching duties. We believe this perception is misguided and based on incorrect interpretations of what scientists are actually doing online. When used in a targeted and streamlined manner, social media tools can complement and enhance a researcher's career. When exploring online tools for the first time, new users can maximize their reach by considering the following points:

**Explore online guides to social media**

- The Superfund program at Oregon State University maintains an exhaustive list of resources (blog articles, videos, how-to guides) focused on science and social media: http://bit.ly/WkdN0G. We recommend this site as a good jumping-off point for new users.

**Establish a professional website (at minimum)**

- To establish an online presence and avoid undesirable Google search results, at minimum researchers should set up a personal website that lays out their specific research projects and areas of expertise, searchable by colleagues, journalists, and the public alike.
- Although professional websites can be established through your university/institute, external hosts (a free site at http://wordpress.com or a custom paid domain) offer more flexibility and are easier to access and maintain.
- If desired, a website can be supplemented with social media accounts (e.g., Twitter and Google+ profiles), which will also appear high in Google search results.

**Locate pertinent online conversations**

- Find people with common interests; follow the social media that they link to and that links to them.
- Use established social networks (e.g., a base of Twitter or LinkedIn contacts) or a means of notification (RSS feeds or personal messages from colleagues/acquaintances) to get started.
- It is completely acceptable to “unfollow” people or groups if their information is not relevant or useful.
- It can be beneficial to read first without contributing (“lurking”) to learn logistics and basic etiquette of different social media platforms.

**Navigate the deluge of online information**

- Strictly maintaining and organizing online accounts is an effective way to filter information (e.g., grouping people using Twitter lists and Google+ circles).
- Similar efficiency can be achieved by tracking and prioritizing the most relevant blogs and articles for reading (e.g., using RSS services such as Google Reader that can be accessed and synced to mobile devices via apps such as MobileRSS).
- Popular content is often heavily reposted and shared; the most important articles and conversations will usually reach you at some point.
- Explore multiple social media tools and related sites/apps for managing online accounts (Box 1). Find ones that you prefer with the appropriate features; consistent use of fewer tools is better than spreading yourself too thin across too many platforms.
- Don't be afraid to ask for help; there are many friendly and established communities who are willing and eager to assist new users.

**Interact with diverse participants**

- Effective social media use *requires* engagement with the audience.
- New users must be open to engaging with people outside one's own professional background or realm of scientific expertise.
- Tone of discussions can vary wildly, from cordial (e.g., conversations about fascinating species) to highly argumentative (e.g., politically sensitive topics such as climate change).
- Users striving to impose a specific viewpoint on their audience (e.g., #arseniclife, http://nbcnews.to/152OCTH) or that are perceived to promote discrimination/sexism (e.g., #womenspace, http://bit.ly/KnEPRy) often face significant backlash and outrage.

**Reach your audience**

- Online communication methods only reach people who are interested in talking about science online.
- Mainstream media continues to represent the most effective platform for disseminating scientific information to broad audiences; 66% of Americans get their news through television, 43% through the internet, 31% through newspapers, and 19% through radio (participants were allowed to name two sources; 2011 Pew poll, http://goo.gl/g2j45).
- Online communities, conversations, and user demographics (sex ratios, racial demographics [15]–[17]) can vary across different tools, with surprisingly little overlap. Using multiple tools may be necessary to achieve one's goals. Notably, many people shy away from using Facebook in light of lingering concerns about privacy (http://nyti.ms/KkwbDE).
- The majority of established bloggers (72% of 126 blogs surveyed [3]) use Twitter as a complementary outlet for disseminating new blog posts to followers.

### Online Tools Improve Research Efficiency

Seasoned internet users are often adamant that online tools can increase their productivity and lead to overall improvements in their personal research efficiency. Unfortunately for data-driven scientists, the majority of present evidence is anecdotal. Twitter has helped busy academics keep up with new research developments, prepare teaching materials, and offer guidance for graduate students (http://bit.ly/VsyERg, http://bit.ly/UTAQ1i, http://bit.ly/VN6hyf). In one extreme case, when faced with a looming deadline for obtaining export permits, Facebook helped researchers identify thousands of fish specimens in under a week [2]. Other researchers use online activities as a way to organize their thoughts and research notes (e.g., online lab books; http://bit.ly/W3f4LL), or to foster creativity and hone their writing skills [3].

Online communities can be especially useful for niche topics where community members have specific needs or require specialized interactions. For example, blog updates and discussion forums can offer user support for software (e.g., programs written in R, http://www.r-bloggers.com), while communities of taxonomists may benefit from a wiki devoted to a particular group of organisms (e.g., the Octopus News Magazine Online for cephalopods, http://www.tonmo.com). Research-focused portals can also result in content curation—amalgamating disparate resources into an organized whole and weeding out untrustworthy sources. Futhermore, citizen science projects (http://www.scistarter.com) and online scientific games (e.g., Foldit for protein structure [4]) assist scientists by allowing members of the general public to make unique and meaningful contributions to ongoing research projects.

The increasing use of online resources may eventually transform and expand the culture of science as a whole. Blogs and social media tools offer an ideal medium for extended scientific conversations (both preprint commentary, such as at http://arXiv.org, and postpublication review) and enable fast-paced discussions of topics that scientists “want and need to discuss” (e.g., topics where peer review is not suitable or necessary [5]; http://bit.ly/WLeajr). It is also increasingly common for blog posts to serve as the basis for peer-reviewed manuscripts (this article, as well as examples cited in [5]). Author Jeremy Fox [5] argues that the online scientific community could become a powerful force for promoting important causes and connecting with policymakers; such impacts have already been seen in the economics community, where blog posts and online discussions led to groundbreaking policy decisions at the US Federal Reserve.

### Online Visibility Helps Track and Improve Scientific Metrics

There is mounting evidence to suggest that an active online presence may directly impact a researcher's credentials as measured through traditional metrics. One UK researcher observed that tweeting and blogging about her own papers led to spikes in the number of article downloads, even for older literature that had been available for years without much previous attention (http://bit.ly/LxpbDz). For articles deposited in the preprint server arXiv, Twitter mentions were positively correlated with rapid article downloads and citations appearing only months after deposition [6]. It is presently unclear as to whether tweeting leads to long-term increases in citations or merely highlights high-quality science that would garner numerous citations even in the absence of social media coverage. However, Eysenbach [7] reported that highly tweeted journal articles were 11 times more likely to be highly cited versus articles without strong social media coverage. Priem et al. [8] additionally demonstrated that journal articles come in drastically different “flavors,” in terms of the way that they are disseminated and consumed among the research community. Social media and article-level metrics may thus be particularly important for unveiling research impacts that cannot be reflected in traditional scientific metrics; for example, Priem et al. noted that some articles may be rarely cited, but heavily read and downloaded by academics.

### Social Media Enhances Professional Networking

Online discussions can lead to tangible, real-world social interactions. Before ever meeting in person, conversations on Twitter can serve as an icebreaker once two people finally meet in a conference or workshop setting. The online world can also broaden a scientist's impact in the research world. Tweeting from conferences (discussing cutting-edge research developments, linking to journal articles or lab websites, e.g., http://bit.ly/11CGRGL) can introduce other scientists to valuable content, and consequently provide networking opportunities for users who actively post during meetings. Because Twitter serves as an information filter for many scientists, publicizing articles on social media can alert researchers to interesting studies that they may not have otherwise come across (e.g., research in journals tangential to their field or within-discipline publications they do not normally read). Journalists and scientists following a conference tweet stream may be additionally introduced to new groups of researchers (particularly early-career scientists or those scientists who are new to Twitter) with relevant and related interests; conference tweeting can thus serve to enhance in-person networking opportunities by expanding these activities to online spheres. For example, a researcher (who asked to remain anonymous) followed HMB and MCG's live tweets from the 2012 Ocean Sciences Meeting and discovered that a scientific question forming the basis of an unsubmitted grant proposal had already been answered. This saved the researcher the effort of submitting a proposal that was unlikely to be funded.

### Broadening “Broader Impacts”

Along with forging links between scientists, online interactions have the potential to enhance “broader impacts” by improving communication between scientists and the general public [9]. An established track record and well-thought-out online outreach strategy can satisfy broader impacts criteria that are increasingly required by funding agencies such as the National Science Foundation. Blogs were being touted as an important outlet for scientists as early as 2006, when researchers were urged to “contribute informed opinions to environmental debates and develop a collective presence in the blogosphere, thereby increasing its inherent credibility” [10]. In some respects, the internet can be a more powerful force than traditional channels—when content goes “viral,” the reach can be truly global. Two projects aimed at changing the perception of science and scientists themselves have recently gone viral in the online science world: the hashtag #iamscience (soon to be turned into a book and podcast) and “This is What a Scientist Looks Like” (http://bit.ly/SayFt2). These initiatives are meant to raise scientists' profiles, dispel ubiquitous stereotypes, and highlight the unconventional career paths followed by most scientists. Such campaigns would be difficult to pursue within the formalized structure of research and academia.

## Defining Goals and Choosing among Online Tools

**Figure 2:**
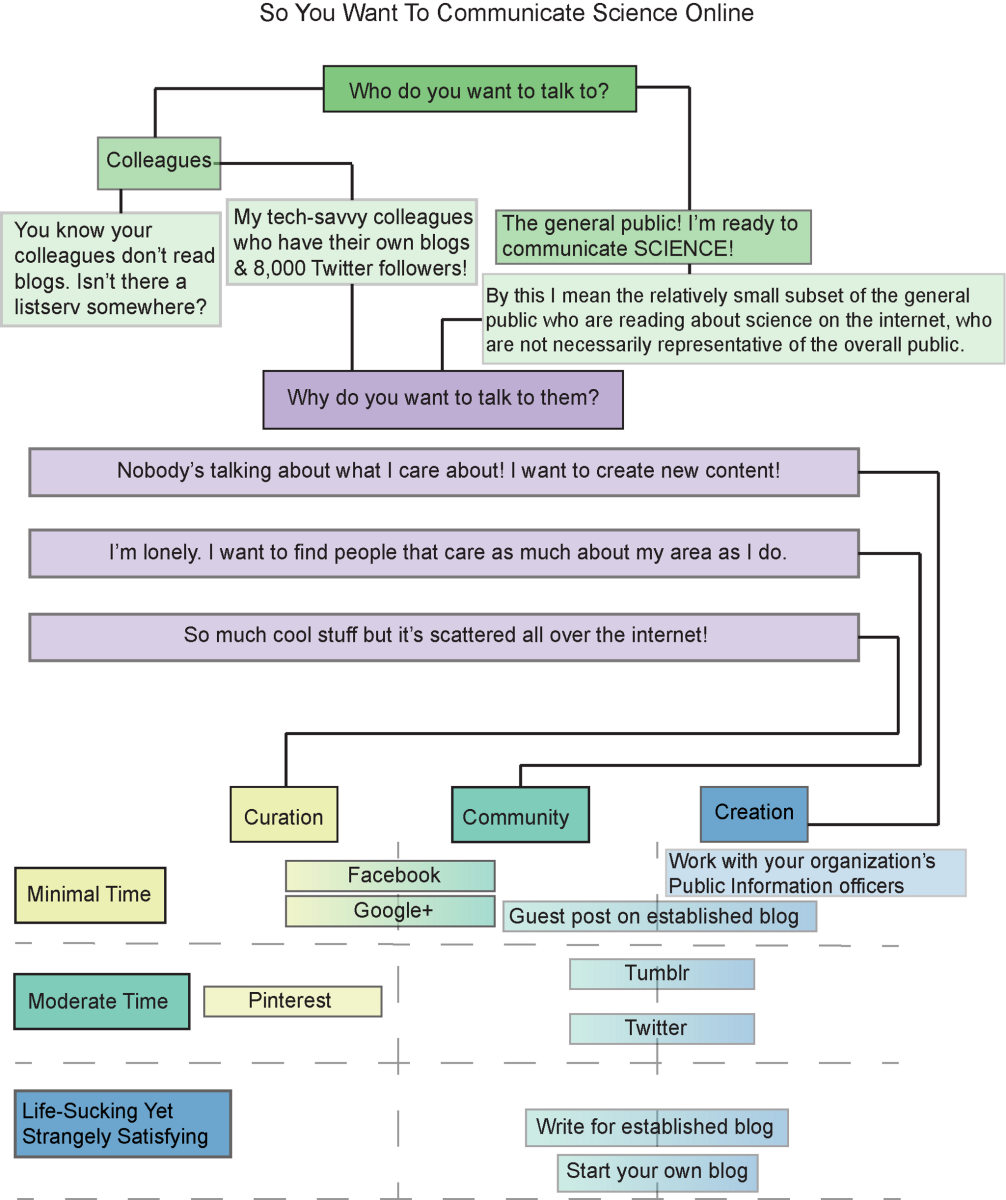
Flowchart showing a decision tree for scientists who are interested in communicating online. An earlier version of this flowchart appeared in a guest post by MCG in *Nature*'s Soapbox Science blog (http://goo.gl/AeKjJ).

**Figure 3:**
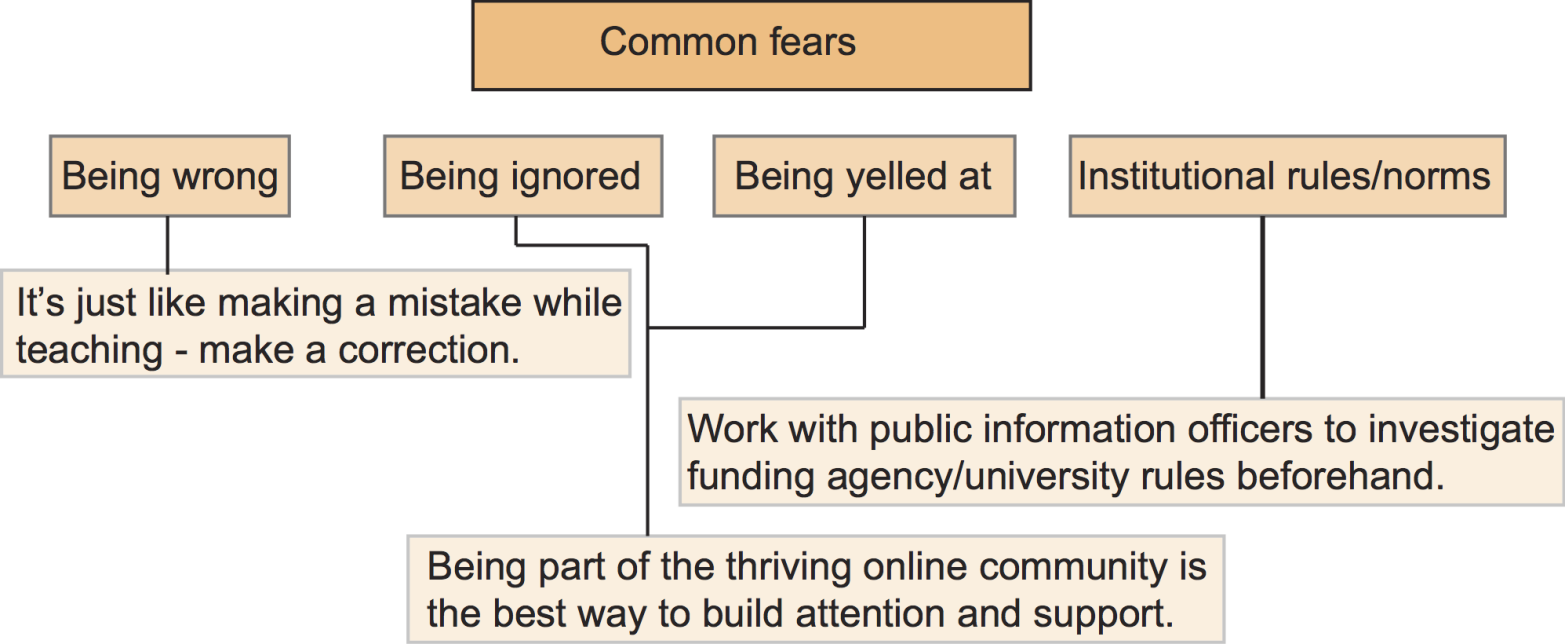
Common online communication fears and suggested solutions. An earlier version of this figure appeared in a guest post by MCG in *Nature's* Soapbox Science blog (http://goo.gl/AeKjJ).

The internet represents an increasingly vast toolbox, and it can be difficult to choose among the long list of “core” resources (Box 1). For those starting out, it is critical to first define what you want to achieve, and then set out to use the tools that are best targeted toward this goal (Figure 2 provides an overview flowchart to help initially define these goals, while Figure 3 lists some common fears for new users); online tools are most effective when customized and used for a specific purpose (http://bit.ly/13J7AAS). Do you want to disseminate information about a discrete event, such as a field expedition? Do you want to build a community of your scientific peers? Do you want to communicate your science to a nonscientist audience? To save time and target the most efficient resources, it is important to think about the timeline of your goals and the time commitment you are willing or able to make. In addition, each social media portal offers unique features, which can complement each other when content is shared between sites.

The next step is to choose online tools that will be maximally beneficial for your specific needs. Blog posts are long form and long-term projects. They require greater initial time investments—crafting and editing posts can take hours—but blog content can be widely disseminated, linked via search engine terms, and provide an “expert” information source that is accessible for years to come. At Deep Sea News, a marine science blog where HMB and MCG are both scientific contributors (http://deepseanews.com), website analytics reveal that most users arrive at the blog via generalized search queries such as “deep sea” and are directed to archived posts with informative content. For example, a January 2011 post entitled “Deep Sea 101: What is the Deep Sea?” is a popular search engine–driven entry point to the blog.

Twitter, on the other hand, is short form and ephemeral—its true appeal lies in the zeitgeist. Twitter users share information and converse in real time, such as through discussions that occur while following a live event (conference talks or workshop discussions tagged with unique keywords, referred to as hashtags; see Box 1) or while remotely participating in a shared activity (e.g., #FridayNightScience, an online outlet for escaping the often-solitary nature of scientific research). Users should note that Twitter itself quickly archives “old” content—for example, tweets amalgamated under a popular conference hashtag may no longer be visible or accessible via searches after a few days. To some extent, using tweet-timing tools (e.g., http://bufferapp.com) can be harnessed to maximize viewership. When Twitter is used correctly, participants should feel that they have an up-to-the-minute personalized news feed and are participating in relevant and meaningful conversations.

Regardless of the platform, social media interactions require two-way conversations (see Box 2). Joining one of the many preexisting scientific conversations can simultaneously disseminate your own content, expand your online network, and raise your professional visibility. An easy entry point is the ScienceOnline conglomerate (http://scienceonline.com), an enthusiastic group of science communicators ranging from tenured professors to freelance journalists [9],[11],[12].

## Long-term Needs and Outlook

Social media and internet-based resources are increasingly ubiquitous. Thus, there is a pressing need for scientific institutions to offer formalized training opportunities for graduate students and tenured faculty alike to learn how to effectively use this new technology. Such training should address common misconceptions about social media platforms and help researchers identify an online repertoire that works best for their specific needs and goals. Organizations such as COMPASS (http://www.compassonline.org) can be called in to offer social media training workshops for scientists, and books such as *Escape from the Ivory Tower* [13] are succinct reference texts offering advice and guidance for interacting with a variety of media sources.

One barrier impacting tool adoption and training opportunities is the fact that online tools are commonly viewed as “uncharted territory.” The novelty of these resources often clouds our understanding of their measurable impacts and long-term utility, particularly in regards to research productivity and science communication/education efforts. In order to understand and refine online tools, appropriate and quantitative metrics are needed. Without high-quality data, it will be impossible to understand the true reach of these tools and discover the most effective uses of different platforms. The altmetrics movement (http://bit.ly/W3gRAD) has sprung up in response to this scenario, aiming to provide a means to measure the true impact of scientific research (social media discussion, journalistic coverage, etc.), as opposed to the perceived value of the venue (e.g., a journal) where research findings may be published. New tools for tracking a researcher's output include Google Scholar profiles (http://scholar.google.com), ImpactStory (http://impactstory.org), and the Open Researcher and Contributor ID (ORCID) initiative (http://orcid.org). In addition, publishers such as PLOS are increasingly offering article-level metrics that log the number of article views, PDF downloads, social media discussions, and associated blog/media coverage.

Social media continues to evolve, grow, and undergo metamorphosis. The use of online tools and cutting-edge technology is growing among scientists, but their adoption and acceptance remains limited across the wider research community. In a 2011 study, only 2.5% of UK and US academics had established a Twitter account [14]. As the benefits become more apparent and dedicated metrics are developed to supplement scientists' portfolios, social media may soon become an integral part of the researcher's toolkit.
